# Chemical Alkaline Leaching and Alkaliphile-Driven Bioleaching: Advancing Metal Recovery from Ores

**DOI:** 10.3390/microorganisms13112577

**Published:** 2025-11-12

**Authors:** Shuang Zhou, Xianglong Qi, Weijian Yu, Qingjun Guan, Yongjie Bu, Jianyu Zhu, Guohua Gu, Tiantao Li, Chenyang Zhang

**Affiliations:** 1 School of Resources, Environment and Safety Engineering, Hunan University of Science and Technology, Xiangtan 411201, China; zhoushuang@hnust.edu.cn (S.Z.); hdqixianglong@mail.hnust.edu.cn (X.Q.); ywjjian886@163.com (W.Y.); guanqingjun@hnust.edu.cn (Q.G.); 1010104@hnust.edu.cn (Y.B.); 2 School of New Energy and Mining, Xinjiang University of Technology, Hotan 848023, China; zhangchenyang@csu.edu.cn; 3 Key Laboratory of Biometallurgy of Ministry of Education, School of Minerals Processing and Bioengineering, Central South University, Changsha 410083, China; zhujy@csu.edu.cn (J.Z.); guguohua@csu.edu.cn (G.G.); 4 State Key Laboratory of Geohazard Prevention and Geoenvironment Protection, Chengdu University of Technology, Chengdu 610059, China

**Keywords:** metal ores, chemical leaching, microbial leaching, alkali-tolerant microorganisms, alkaline microbial leaching

## Abstract

Ore leaching constitutes a core step for achieving efficient utilization of mineral resources, primarily encompassing acid leaching and alkaline leaching methods. Currently, acid leaching technology has reached a high level of maturity and is widely applied in industry due to its advantages of fast reaction kinetics and broad applicability to various mineral types. However, the theoretical framework underpinning alkaline leaching systems remains relatively weak. Given the distinct advantages of alkaline leaching in processing ores containing alkaline gangue minerals, this review systematically examines chemical and microbial leaching techniques for metal ores under alkaline conditions. It focuses on elucidating the mechanisms and key influencing factors associated with different alkaline matrices, oxidants, external pressures, and microbial strains. Future development prospects are also discussed. The aim is to provide a theoretical foundation and practical guidance for advancing metal ore leaching technologies towards greener and more efficient directions.

## 1. Introduction

Within the field of metallic mineral resource processing, the dual challenges of increasingly severe resource scarcity and environmental pollution intensify, rendering the advancement of efficient and environmentally friendly resource utilization technologies a core imperative for the industry [1]. Hydrometallurgical technology has demonstrated significant application value in treating complex refractory ores and efficiently reclaiming secondary resources, positioning it as a crucial technological approach to address this challenge [2]. Acidic chemical leaching systems are widely employed due to their high extraction efficiency for various metals. Concurrently, microbial metallurgical techniques under acidic conditions have garnered considerable interest owing to their low energy consumption and environmental compatibility [3]. Presently, the traditional acid leaching process has achieved industrial-scale application in the extraction of metals such as copper and gold. While the industrial application scope of microbial metallurgy remains relatively limited, significant progress has been made in understanding relevant microbial physiology, elucidating reaction mechanisms and optimizing processes.

For ores and secondary resources containing alkaline gangue minerals or amphoteric metals, alkaline leaching systems exhibit unique potential advantages. These systems leverage the synergistic action of alkaline media and oxidizing agents to achieve selective complexation–dissolution of target metals [4]. Simultaneously, they exploit the inherent chemical inertness and surface passivation effect of alkaline gangue within the system to significantly suppress its dissolution. This strategy circumvents issues prevalent in acidic systems, such as high acid consumption and colloid formation. However, fundamental theoretical research on alkaline chemical leaching has yet to establish a comprehensive framework, and its process optimization lags behind the well-developed acidic systems. Similarly, the research foundation for alkali-tolerant microbial leaching is relatively weak. Currently identified alkali-tolerant bioleaching microorganisms are limited in diversity, and understanding of their leaching efficacy, mechanisms of action, and community ecology remains insufficient [5]. In recent years, the global mineral supply landscape has undergone a significant transformation. Easily processable sulfide resources are gradually being depleted, while low-grade oxidized and alkaline ores have emerged as critical future sources of metal supply. Traditional acidic leaching processes face severe challenges when treating such ores, including excessive reagent consumption, high co-dissolution of impurities, and pronounced environmental risks [6]. Consequently, the development of green leaching technologies tailored to the inherent characteristics of alkaline ores is of immediate practical importance. Such technologies are crucial for ensuring the sustainable supply of global metal resources and for reducing the overall environmental footprint of extractive metallurgy. Alkaline chemical and bioleaching technologies, leveraging their intrinsic selectivity, lower reagent consumption, and superior environmental compatibility, offer a highly promising solution for the efficient utilization of these refractory resources. This potential has established alkaline leaching as a key strategic focus for breakthrough innovation in the field of international biohydrometallurgy. Therefore, promoting the development of alkaline leaching technology holds profound strategic significance for expanding the boundaries of biohydrometallurgy and realizing the green exploitation of mineral resources.

This review systematically analyzes the multi-modal action mechanisms of media, oxidizing agents and pressure within alkaline chemical leaching. It further clarifies the molecular principles and application progress of microbial leaching under alkaline conditions. The overarching aim is to establish a theoretical foundation and an engineering reference framework for research on alkaline systems.

## 2. Alkaline Chemical Leaching

Alkaline chemical leaching employs alkaline media as lixiviants to selectively transfer target metals from minerals into the liquid phase through coordination dissolution or redox reactions. Compared to conventional acidic systems, it possesses significant environmental advantages, such as reduced consumption of strong acids, diminished challenges in waste solution treatment and lower corrosion risks [7]. The core efficacy of this technology is governed by the synergistic interplay of the alkaline matrix, oxidant type and pressure conditions. Consequently, systematically analyzing the independent mechanisms and synergistic enhancement pathways of these parameters is crucial for optimizing process carbon intensity and reducing capital expenditure [8].

### 2.1. Alkaline Matrix

The alkaline matrix refers to substances within the leaching system possessing proton-accepting or electron-donating capabilities, functioning as the reaction medium or direct ligands. Its selection directly impacts the thermodynamic equilibrium and kinetic rate of metal dissolution. Common alkaline matrices include sodium hydroxide/potassium hydroxide, glycine, sodium sulfide, and sodium hypochlorite.

#### 2.1.1. Sodium Hydroxide or Potassium Hydroxide

**Figure 1:**
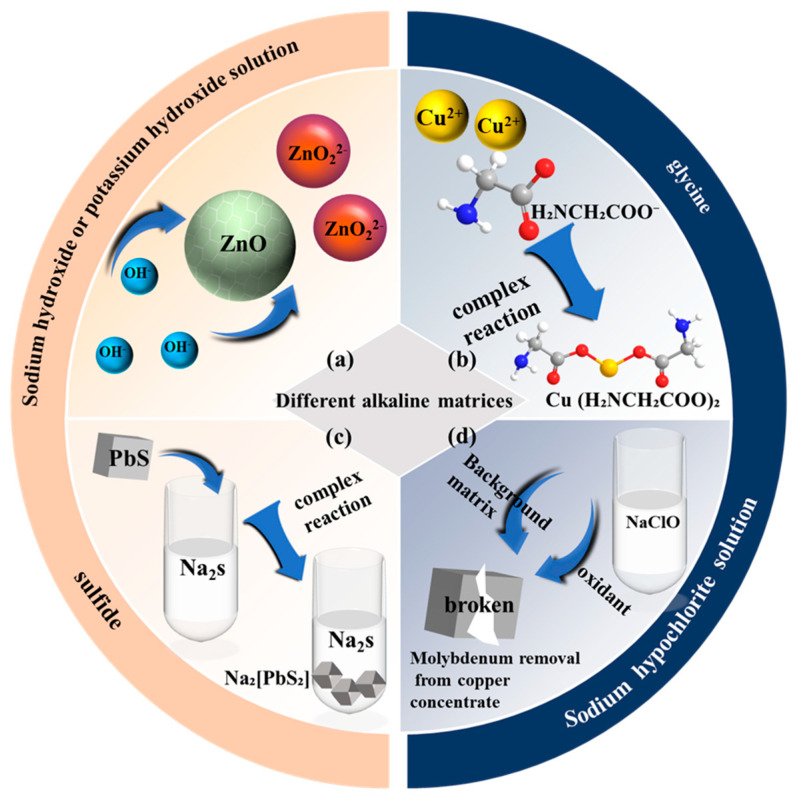
Different alkaline matrices and their leaching mechanisms (**a**–**d**). Mechanisms of zinc oxide ore leaching by sodium hydroxide or potassium hydroxide (**a**); Cuprite leaching by glycine (**b**); Lead-zinc ores leaching by sodium sulfide (**c**); Molybdenum removal from copper concentrates by sodium hypochlorite (**d**).

Sodium hydroxide (NaOH) and potassium hydroxide (KOH) serve as strong alkaline leaching matrices. Their mechanism relies on the synergistic action of OH^−^ nucleophilic attack and Lewis base coordination. In the leaching of zinc oxide ore (ZnO) (Figure 1a), NaOH attacks the Zn-O bond in the ZnO lattice via nucleophilic substitution, generating soluble sodium zincate (Na_2_ZnO_2_) (Equation (1)), thereby achieving zinc dissolution [9]. Metallic zinc of high purity can be directly recovered from a purified sodium zincate (Na_2_ZnO_2_) solution via electrolytic deposition. In this process, the solution is electrolyzed in a cell using stainless steel as both the cathode and the anode. Under an applied voltage, zinc ions are reduced at the cathode to form metallic zinc powder with a purity exceeding 99.5%. The spent electrolyte, which consists primarily of NaOH, can be recirculated to the leaching stage, thereby forming a closed-loop process that significantly reduces alkali consumption and wastewater discharge. For stibnite (Sb_2_S_3_) systems, KOH exhibits unique advantages. OH^−^ cleaves Sb-S bonds, forming oxythioantimonate (SbOS^−^) and thioantimonite (SbS_2_^−^) ions. Concurrently, the potassium ion (K^+^) significantly enhances the solubility of the Sb-O/Sb-S complexes due to a cation chelation effect attributed to its ionic radius compatibility (matching the anionic cavity structure), low charge density (reducing electron cloud repulsion) and weak hydration characteristics (lowering desolvation energy barriers). This effect establishes a concentration gradient driving force based on Le Chatelier’s principle, continuously shifting the dissolution equilibrium forward, thus promoting the efficient decomposition of stibnite [10] (Equation (2)). In the KOH leaching of antimony ore, the resulting leachate can subsequently be processed into valuable antimony compounds through chemical precipitation. In this method, H_2_O_2_ and NaCl are added to the leachate, and the mixture is reacted at a controlled temperature to form a precipitate of sodium pyroantimonate (NaSb(OH)_6_). After filtration, washing, and drying, the sodium pyroantimonate product can be directly utilized as a flame retardant, ceramic additive, or semiconductor precursor, thereby achieving efficient resource recovery of antimony.

The primary merits of these strong alkaline lixiviants stem from their high reactivity and rapid leaching kinetics, chiefly manifested in the efficient nucleophilic attack capability of OH^−^ on mineral lattices and the stability of Lewis base coordination. However, at elevated concentrations, the risk of electrochemical corrosion and stress corrosion cracking induced by strong alkaline media rises substantially. This necessitates the use of specialized corrosion-resistant materials for critical equipment like reactors and pipelines, increasing capital investment compared to standard systems. Maintenance also requires addressing susceptibility to intergranular corrosion. More critically, the residual strong alkaline effluent post-leaching demands multi-stage treatment processes including acidification precipitation, membrane separation and neutralization flocculation, leading to increased overall treatment costs and severely constraining industrial economic viability.

ZnO + 2NaOH = Na_2_ZnO_2_ + H_2_O (1)

Sb_2_S_3_ + 2KOH → KSbOS + KSbS_2_ + H_2_O (2)

#### 2.1.2. Glycine

Glycine (H_2_NCH_2_COOH), an amphoteric organic ligand, undergoes deprotonation under alkaline conditions (pH > 10.5) to form the glycinate ion (NH_2_CH_2_COO^−^). This ion acts as a bidentate chelating agent, utilizing its amino nitrogen and carboxylate oxygen atoms to form highly stable complexes with metal ions, thereby facilitating metal dissolution. Within the cuprite (Cu_2_O) system, glycine mediates a stepwise dissolution mechanism. The cuprite lattice dissociates, releasing Cu^+^ ions. These dissolved Cu^+^ ions are subsequently oxidized to Cu^2+^, which then coordinates with two NH_2_CH_2_COO^−^ ligands to form the electrically neutral tetrahedral complex [Cu(NH_2_CH_2_COO)_2_], enabling efficient copper transfer (Figure 1b and Equation (3)) [11]. Copper metal of high purity can be directly recovered from a leaching solution containing the copper-glycine complex by electrolysis, where it is deposited at the cathode through electrochemical reduction. In the leaching of low-sulfide oxidized gold ores, a synergistic glycine-potassium permanganate (KMnO_4_) system dissolves gold through a coupled oxidation-complexation approach. Potassium permanganate oxidizes elemental gold (Au^0^) to Au(I)/Au(III) states. Simultaneously, NH_2_CH_2_COO^−^ forms a linear bidentate complex [Au(NH_2_CH_2_COO)_2_]^−^ with the gold ions, effectively suppressing their reprecipitation (Equations (4) and (5)). The alkaline environment (pH > 10.5) is a critical control parameter. It enhances the deprotonation degree of glycine and elevates the electrochemical potential of the oxidant (KMnO_4_), synergistically optimizing both complexation efficiency and oxidation kinetics. Gold-glycine complexes in the pregnant solution can be effectively adsorbed onto activated carbon derived from coconut shells. Under the tested conditions, the loaded carbon achieved gold loadings of 610 ppm and 596 ppm in the *Carbon-in-Leach* (CIL) and *Carbon-in-Pulp* (CIP) configurations, respectively. The precious metal was subsequently recovered from the carbon through conventional elution and electrowinning processes, producing gold of high purity [12].

The selective leaching advantage of glycine for copper and gold stems from its high coordination selectivity coefficient. This property significantly inhibits the dissolution of high-charge-density impurity ions like Fe^3+^ and Al^3+^, due to distinct differences in chelation energy [13]. This impurity suppression mechanism prevents issues in subsequent processing stages, such as third-phase formation during solvent extraction, organic phase poisoning, and impurity-induced electrode passivation in electrowinning, thereby markedly enhancing process stability [14,15]. Furthermore, glycine’s inherent low cost and biodegradable nature contribute to reducing overall operational costs while aligning with green metallurgical standards.

Cu^2+^ +2H_2_NCH_2_COO^−^ = Cu(H_2_NCH_2_COO)_2_ (3)

MnO_4_^−^ + 2H_2_O + 3e^−^ → MnO_2_↓ + 4OH^−^ (4)

4Au + 8NH_2_CH_2_COO^−^ + O_2_ + 2H_2_O → 4[Au(NH_2_CH_2_COO)_2_]^−^ + 4OH^−^ (5)

#### 2.1.3. Sodium Sulfide

Sodium sulfide (Na_2_S) serves as an effective leaching agent for processing complex sulfide minerals containing lead, antimony, tellurium and similar elements. Its fundamental advantage lies in achieving selective dissolution of target metals through the precise modulation of sulfur anion coordination chemistry. During the leaching of lead-zinc ores (where galena, PbS, is the predominant phase), Na_2_S undergoes a thiol ligand exchange reaction with PbS (Figure 1c and Equation (6)), forming the soluble complex sodium dithiolate(II) (Na_2_[PbS_2_]). The chelating action of the [S_2_]^2−^ ligand facilitates lead dissolution into the aqueous phase. For tetrahedrite-rich concentrates containing copper and antimony undergoing alkaline leaching, Na_2_S preferentially dissolves antimony via stereoselective coordination ligands, generating soluble thioantimonite species (NaSbS_2_) (Equation (7)). Concurrently, copper remains sequestered in the solid phase as thermodynamically stable chalcocite (Cu_2_S), enabling efficient antimony-copper separation [16]. In the treatment of tellurium-rich antimony residues, Na_2_S employs an oxidative sulfolysis mechanism. This converts tellurium and antimony into sodium tetrathiotellurate (Na_2_TeS_4_) and sodium tetrathioantimonate (Na_3_SbS_4_), respectively (Equations (8) and (9)). Simultaneously, impurity metals such as lead, bismuth, iron, and zinc are immobilized within the residue as insoluble sulfide precipitates, establishing a foundation for the co-recovery of tellurium and antimony. Elemental tellurium can be selectively precipitated from the alkaline leaching solution containing Na_2_TeS_4_ by adding sodium sulfite (Na_2_SO_3_) as a reducing agent. Subsequently, sodium antimonate (NaSb(OH)_6_) is precipitated from the same solution through the addition of hydrogen peroxide as an oxidant, thereby achieving the sequential recovery of tellurium and antimony. Critically, the leaching kinetics of Te/Sb from high-tellurium residues are governed by Na_2_S concentration diffusion control. Within the 0–40 g/L range, extraction efficiency increases with rising concentration. Beyond a critical threshold,; however, interfacial mass transfer saturation leads to increment attenuation [17].

The preeminent advantage of sodium sulfide leaching resides in its ligand field selectivity. By manipulating the coordination number and oxidation state of sulfur anions, it achieves targeted extraction of lead, antimony and tellurium. Crucially, it exploits the inherent differential solubility product (Ksp) of sulfide compounds to efficiently suppress the dissolution of impurity metals (Pb, Bi, Fe, Zn). This selectivity significantly reduces impurity loading within the pregnant leach solution while enhancing impurity metal concentration within the leach residue. Consequently, it substantially streamlines subsequent downstream separation processes [18].

PbS + Na_2_S = Na_2_[PbS_2_] (6)

Cu_12_Sb_4_S_13_ + 2Na_2_S ⟶ 5Cu_2_S + 2CuS + 4NaSbS_2_ (7)

Na_2_TeO_4_ + 4Na_2_S + 4H_2_O ⟶ Na_2_TeS_4_ + 8NaOH (8)

NaSb(OH)_6_ + 4Na_2_S ⟶ Na_3_SbS_4_ + 6NaOH (9)

#### 2.1.4. Sodium Hypochlorite

Sodium hypochlorite (NaClO) demonstrates dual synergistic functionality as a strongly oxidizing alkaline medium in refractory ore leaching. It sustains a high-pH environment (pH 10–12) to inhibit hydrolysis of primary metals while enabling selective oxidation of target metals via hypochlorite ions (ClO^−^). In molybdenum removal from copper concentrates, NaClO oxidizes molybdenum to high valence state (Mo^6+^), generating soluble sodium molybdate (Na_2_MoO_4_) (Figure 1d and Equation (10)). Concurrently, copper dissolution is inhibited due to the formation of a passivation layer (CuO/Cu(OH)_2_). Molybdenum leaching kinetics are governed by NaClO concentration, NaOH concentration, temperature and liquid-to-solid ratio [19]. Analogously, during arsenic removal from tennantite (Cu_12_As_4_S_13_), ClO^−^ cleaves As–S bonds through nucleophilic attack, driving arsenic’s valence transition from As^3+^ to As^5+^ to form soluble arsenate ions (AsO_4_^3−^). Simultaneously released Cu^2+^/Fe^3+^ ions precipitate as Cu(OH)_2_ and Fe(OH)_3_ under alkaline conditions, achieving solid–liquid separation of arsenic from copper/iron [20]. For gold-bearing enargite (Cu_3_AsS_4_), ClO^−^ oxidizes arsenic to hydrogen arsenate (HAsO_4_^2−^) (Equation (11)), while copper transforms into thermodynamically stable CuO, co-depositing with unreacted gold (Au^0^) in the residue. This accomplishes dual objectives of arsenic elimination and precious metal enrichment [21].

Hypochlorite exhibits exceptional selectivity in processes such as molybdenum extraction and arsenic removal, significantly minimizing primary metal losses [22]. Its principal advantage lies in efficiently oxidizing arsenic-bearing minerals that resist conventional acid/base treatment. However, NaClO incurs relatively high costs and suffers from instability with decomposition tendencies. Consequently, excess reagent addition is typically required, alongside precise control of concentration, pH, temperature, and light exclusion to preserve effective oxidizing capacity.

MoS_2_ + 9NaClO + 6NaOH = Na_2_MoO_4_ + 2Na_2_SO_4_ + 9NaCl + 3H_2_O (10)

2Cu_3_AsS_4_ + 14ClO^−^ + 10OH^−^ = 6CuO + 2HAsO_4_^2−^ + 4SO_4_^2−^ + 14Cl^−^ + 4H_2_O (11)

### 2.2. Oxidants

The selection of oxidants constitutes a pivotal strategy to enhance leaching kinetics and optimize resource utilization efficiency within leaching processes. Commonly employed oxidants such as hydrogen peroxide (H_2_O_2_), ozone (O_3_), oxygen (O_2_), and potassium permanganate (KMnO_4_) exert significant effects in alkaline leaching systems through divergent oxidation pathways, encompassing direct electron transfer or radical-mediated mechanisms.

#### 2.2.1. Hydrogen Peroxide

**Figure 2:**
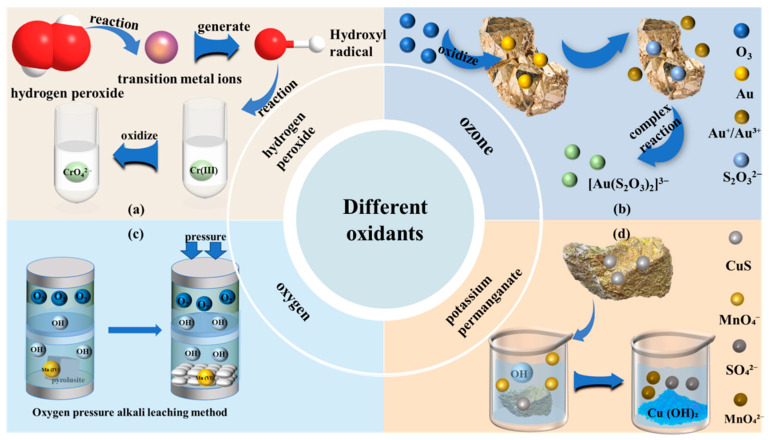
Different oxidants and their leaching mechanisms (**a**–**d**). Mechanisms of chromium-containing slag leaching by hydrogen peroxide (**a**); Gold leaching from pyrite by ozone (**b**); High-pressure alkaline leaching of manganese ore with oxygen (**c**); Copper sulfide ore leaching by potassium permanganate (**d**).

Hydrogen peroxide (H_2_O_2_) demonstrates a dual-pathway oxidation mechanism within alkaline media (pH > 10), enabling an efficient approach for the resource recovery of chromium-containing slag through the synergistic interplay of direct electron transfer and radical-mediated oxidation. In the direct oxidation pathway, hydroperoxyl ions (HOO^−^), formed via the dissociation of H_2_O_2_ (Equation (12)), directly attack Cr(III) within the crystal lattice (primarily FeCr_2_O_4_ spinel phase) of vanadium-chromium slag. This electron transfer process triggers the release of lattice distortion energy, causing structural collapse of the spinel and facilitating chromium dissolution as chromate ions (CrO_4_^2−^). Concurrently, the indirect pathway employs transition metal-mediated Fenton-like reactions to generate hydroxyl radicals (·OH) (Equation (13)), which efficiently oxidize liberated Cr(III) in solution, establishing a secondary oxidation cycle. These pathways form a cascade reaction chain: direct oxidation disrupts the lattice to release Cr(III), followed by deep oxidation of the solubilized chromium species via indirect oxidation [23] (Figure 2a). Crucially, the alkaline environment not only suppresses the unproductive decomposition of H_2_O_2_ but also counteracts the diminished oxidizing capacity typically associated with elevated pH through the high oxidation potential of the generated radicals, resulting in an efficient synergistic system for high chromium recovery [24]. This mechanism extends to alkaline glycinate (Gly^−^) systems for gold leaching, where H_2_O_2_ oxidizes Au^0^ to Au^+^ via a two-electron transfer, while Gly^−^ concurrently forms a stable chelate complex ([Au(Gly)_2_]^+^). The addition of exogenous Cu^2+^ further enhances gold dissolution by catalyzing the formation of ·OOH/·OH radical cycles [25].

Compared to conventional oxidants, H_2_O_2_ offers distinct environmental advantages. Its oxidation end-product is solely water, eliminating concerns about secondary pollution. Reactions proceed effectively under mild conditions, significantly reducing energy consumption compared to high-temperature and high-pressure systems. Furthermore, the reaction medium approaches neutrality, lowering equipment corrosion rates. Given the potential of H_2_O_2_ in green metallurgy, the development of sustainable production methods is gaining significant attention. Among emerging green synthesis routes, the electrocatalytic synthesis of hydrogen peroxide is particularly promising. This method utilizes electrochemical reactions, driven by renewable energy sources such as solar power, to directly generate H_2_O_2_ in solution via the two-electron oxygen reduction reaction (Equations (14) and (15)). This environmentally benign and highly effective oxidation technology provides an economically viable solution for the detoxification and resource utilization of chromium-bearing slags, offering key technical support for the low-carbon recovery of strategic metals [26].

H_2_O_2_ + OH^−^ ⇌ HOO^−^ + H_2_O (12)

Fe^2+^ +H_2_O_2_ → Fe^3+^ +·OH + OH^−^ (13)

O_2_ + 2H^+^ +2e^−^ → H_2_O_2_ (Acid medium) (14)

O_2_ + H_2_O + 2e^−^→HO_2_^−^ + OH^−^ (Alkaline medium) (15)

#### 2.2.2. Ozone

Ozone (O_3_), characterized by its high oxidation potential and environmentally friendly attributes, emerges as a highly promising oxidant in alkaline leaching systems, particularly demonstrating exceptional selectivity in the recovery of metals such as gold and uranium. Under alkaline conditions, ozone, functioning as a potent oxidant (oxidation potential of 2.7 V), oxidizes and disrupts the pyrite lattice structure, liberating encapsulated gold particles. Subsequently, in the presence of thiosulfate (from added Na_2_S_2_O_3_), ozone oxidizes the liberated elemental gold (Au^0^) to Au^+^ or Au^3+^ (Equation (16)). The oxidized gold ions then form a stable, water-soluble complex ([Au(S_2_O_3_)_2_]^3−^) with the thiosulfate ligand (S_2_O_3_^2−^) (Figure 2b). The alkaline environment concurrently inhibits thiosulfate decomposition, maintaining a high concentration of the complexing agent and ensuring the sustained forward progression of the gold complexation reaction. After the successful leaching of gold as [Au(S_2_O_3_)_2_]^3−^ complexes into the thiosulfate solution, the subsequent downstream processing for gold recovery involves several key steps. The pregnant leach solution is first subjected to solid–liquid separation to remove suspended solids. Subsequently, the clarified solution is treated by activated carbon adsorption followed by electrowinning. In this process, the gold-thiosulfate complexes are adsorbed onto the carbon. Following this, the loaded carbon is eluted to generate a concentrated gold solution. This solution is finally fed into an electrolytic cell for electrowinning, resulting in the deposition of high-purity gold onto the cathode [27]. In uranium ore leaching systems employing a carbonate-bicarbonate matrix, ozone significantly enhances uranium extraction efficiency, outperforming methods utilizing air or pure oxygen gas. The mechanism of ozone-mediated mineral oxidation involves a synergistic interplay of direct and indirect pathways. Research indicates ozone can directly oxidize tetravalent uranium (U^4+^) within the ore to hexavalent uranium (U^6+^) via direct electron transfer (Equation (17)), enabling its complexation with carbonate to form soluble uranyl carbonate ions. Indirect oxidation occurs when ozone decomposition, facilitated by alkaline conditions or catalysts (such as transition metal ions Fe^2+^ or Mn^2+^), generates hydroxyl radicals (•OH). These radicals non-selectively oxidize uranium encapsulated within the mineral matrix or reductants tightly bound to uranium (e.g., sulfides, organic matter), thereby liberating the uranium and making it more accessible for subsequent direct oxidation and dissolution by ozone, further promoting uranium extraction [28]. The addition of H_2_O_2_ to concentrated uranyl solutions, under a controlled pH range, results in the precipitation of uranium peroxide (UO_4_·xH_2_O). This approach enables the production of high-purity solid products under mild conditions, such as near room temperature and neutral pH. It thereby offers an environmentally benign alternative to conventional precipitation routes that use ammonium or sodium reagents, as it avoids the introduction of secondary pollutants [29].

A key environmental advantage of ozone is that its final reduction product is oxygen gas, eliminating the introduction of problematic impurities like heavy metal ions or halogens and avoiding secondary pollution challenges common with conventional oxidants. Furthermore, in alkaline systems, ozone decomposition generates reactive species such as the superoxide radical (O_2_•^−^) and hydroperoxyl radical (HO_2_•), which can participate in additional oxidative cycles (e.g., O_3_ + OH^−^ → O_2_•^−^ + HO_2_•), enhancing oxidant utilization efficiency. This attribute aligns well with the principles of green metallurgy [30]. The combination of high selectivity, effective mineral breakdown, enhanced complexation stability in alkali, and minimal secondary waste generation underscores ozone’s significant potential for sustainable metal recovery processes.

Au + 1/2O_3_ + H^+^→Au^+^ +1/2O_2_ + 1/2H_2_O (16)

UO_2_ + O_3_ + 3CO_3_^2−^ + H_2_O→[UO_2_(CO_3_)_3_]^4−^ + 2OH^−^ + O_2_ (17)

#### 2.2.3. Oxygen

Oxygen (O_2_) serves as a significant oxidant in mineral processing due to its low operational cost and ecological benefits. In manganese ore leaching, oxygen pressure alkaline leaching operates as a green, low-carbon metallurgical process. Research conducted within nickel-based alloy high-pressure reactor systems (Figure 2c) demonstrates efficient conversion of manganese ore (containing MnO_2_) into soluble potassium manganate (K_2_MnO_4_) when utilizing potassium hydroxide (KOH) as the alkaline matrix and O_2_ as the oxidant over extended reaction periods (Equation (18)) [31]. Furthermore, this technique exhibits applicability for arsenic removal from arsenic-bearing residues. Under pressurized oxidizing conditions, elevated oxygen partial pressure sustains a strong oxidizing potential. This drives the oxidative decomposition of sulfide minerals within the residue, such as pyrite (FeS_2_), liberating Fe^3+^ into the aqueous phase (Equation (19)). Subsequently, in the strongly alkaline environment, Fe^3+^ undergoes hydrolysis to form hydroxide precipitates, while the oxidized arsenic remains in solution as arsenate ions (AsO_4_^3−^) (Equation (20)). This synergistic process, involving the oxidative dissolution of arsenic and concurrent precipitation of impurity metals, achieves efficient separation while circumventing the arsenic volatilization risks inherent in acidic systems [32].

The oxygen pressure alkaline leaching method offers substantial economic advantages. The utilization of low-cost oxygen gas as an oxidant reduces reliance on expensive chemical reagents typically employed in conventional processes. Emission of harmful gases is eliminated during the reaction, aligning with green chemistry principles. Moreover, optimized conditions enable exceptionally high manganese conversion rates, maximizing resource utilization efficiency.

2MnO_2_ + 4KOH + O_2_ = 2K_2_MnO_4_ + 2H_2_O (18)

4FeS_2_ + 15O_2_ + 4OH^−^ + 2H_2_O ⟶ 4Fe^3+^ +8SO_4_^2−^ + 4H^+^ (19)

FeAsO_4_ + 3OH^−^ ⟶ Fe^3+^ +AsO_4_^3−^ + H_2_O (20)

#### 2.2.4. Potassium Permanganate

In alkaline media, potassium permanganate (KMnO_4_) facilitates the efficient dissolution and enrichment of copper from copper sulfide ores, leveraging its high oxidation potential synergistically with hydroxide ions (OH^−^). The dissociated permanganate ion (MnO_4_^−^) oxidizes copper sulfide (CuS) to sulfate (SO_4_^2−^) and cupric ions (Cu^2+^) (Equation (21)). The released Cu^2+^ ions subsequently combine with OH^−^ to form the thermodynamically stable precipitate copper hydroxide (Cu(OH)_2_), leading to solid-phase enrichment (Figure 2d). This mechanism extends to low-sulfur oxidized gold ores processed in glycine-based systems. Here, KMnO_4_ drives gold dissolution through a dual-path reaction: MnO_4_^−^ directly oxidizes elemental gold (Au^0^) to Au(I)/Au(III) states, concomitant with its reduction to solid manganese dioxide (MnO_2_). Simultaneously, MnO_4_^−^ oxidizes the glycinate anion (NH_2_CH_2_COO^−^), also resulting in co-precipitation as MnO_2_ (Equation (22)). These MnO_2_ particles activate surface water molecules to generate highly reactive hydroxyl radicals (·OH), significantly accelerating the decomposition rate of gold-bearing inclusions and thereby exposing gold particles. The dissolved Au(I) ions then form exceptionally stable complexes with glycine ([Au(NH_2_CH_2_COO)_2_]^−^) (Equation (23)). Gold can be recovered directly from the leachate through a process involving adsorption onto activated carbon, followed by electrolysis [12].

A critical advantage of the alkaline environment (pH > 10) lies in confining the reduction product of MnO_4_^−^ to the solid phase as MnO_2_. Compared to the generation of soluble Mn^2+^ ions in acidic systems, this pathway provides dual suppression. It mitigates interference from soluble manganese species during subsequent metal recovery processes and minimizes the ineffective decomposition of the oxidant itself. This characteristic enhances both process selectivity and oxidant utilization efficiency within the alkaline leaching regime [33].

CuS + 2MnO_4_^−^ + 2OH^−^ = Cu(OH)_2_↓ + 2MnO_4_^2−^ + SO_4_^2−^ (21)

2MnO_4_^−^ + 2(NH_2_CH_2_COO)^−^ + 1/2O_2_

→ 2MnO_2_↓ + 2C_2_O_4_^2−^ + 2NH_3_ + H_2_O (22)

Au + 2MnO_4_^−^ + 4(NH_2_CH_2_COO)^−^ + O_2_

→ Au(NH_2_CH_2_COO)_2_ + 2MnO_2_↓ + 2C_2_O_4_^2−^ + 2NH_3_ + 2OH^−^ (23)

### 2.3. Pressurized Operation

Pressurized operation enhances the efficiency of alkaline hydrometallurgy through synergistic thermodynamic and mass transfer mechanisms. The adiabatic heating effect induced by pressure significantly increases molecular kinetic energy, thereby accelerating the effective collision frequency between minerals and leaching agents and boosting metal dissolution kinetics [34]. Concurrently, according to Henry’s Law, elevated pressure increases the solubility of oxidizing gases, shifting the reaction equilibrium at the gas–liquid–solid interface towards metal ion dissolution and substantially promoting leaching efficiency [35]. However, excessively high pressure triggers dual adverse effects. Firstly, intensified pressure reduces the activation energy for impurity phase reactions, leading to reagent overconsumption. Secondly, it induces surface passivation phenomena. Studies reveal that during the pressurized leaching of magnesia-bearing ores and chalcopyrite, the formation of magnesium hydroxide precipitates and sulfur layers on the mineral surface under excessive pressure impedes further contact between the leaching agents and the ore matrix, resulting in decreased extraction yields [36]. Furthermore, high-pressure environments necessitate the use of high-strength materials for reactors, increasing capital investment costs, while the risk of sealing failure escalates exponentially with pressure. Consequently, precise modulation of the pressure window becomes paramount for balancing leaching intensification, maximizing oxidant utilization efficiency and controlling life-cycle costs.

## 3. Bioleaching

Bioleaching technology demonstrates distinct advantages over conventional metallurgical processes in processing low-grade ores [37]. This approach leverages the biocatalytic activity of microorganisms to operate effectively under ambient pressure conditions, thereby eliminating the need for high-temperature and high-pressure equipment. This fundamental shift significantly reduces energy consumption and associated capital costs [38]. Furthermore, its inherent environmental compatibility stems from the efficient natural degradation pathways of microbial metabolites. This characteristic leads to a measurable decrease in the toxicity leaching concentration of tailings, thereby significantly cutting down waste disposal expenditures. The core drivers of these advantages reside in the biochemical metabolic pathways engineered within functional leaching strains. Based on their optimal growth pH gradients, these microorganisms are systematically classified into two primary categories including acidophilic microbes and alkali-tolerant microbes [39].

### 3.1. Acidophilic Microorganisms and Bioleaching Mechanisms

*Acidophilic* bioleaching microorganisms, functioning as specialized communities within extreme acidic environments (pH 0.5–3.0), are widely distributed in acid mine drainage (AMD), sulfide ore deposits, and geothermal regions. They possess evolutionarily adapted tolerance mechanisms for high H^+^ concentrations and heavy metal ions [40]. Utilizing chemolithoautotrophic metabolism to oxidize inorganic substrates (Fe^2+^/reduced sulfur compounds) for energy transduction, these microorganisms demonstrate significant potential for efficient metal dissolution in biohydrometallurgy [41]. Based on their primary energy metabolism pathways, they can be classified into three categories including iron- and sulfur-oxidizing bacteria, obligate iron-oxidizing bacteria and sulfur-oxidizing bacteria [42]. As the primary source of global nickel, laterite ores often contain cobalt as an associated valuable element. However, nickel and cobalt are frequently locked within stable mineral phases, such as goethite, cobalt arsenites, and serpentine, via isomorphic substitution or surface adsorption. Conventional pyrometallurgical processes are often economically unviable for such low-grade ores due to their high energy consumption, operational costs, and environmental impact. In contrast, *acidophilic* leaching microorganisms, with their exceptional acid tolerance, chemoautotrophic acid-generating capability, and redox regulation functions, offer a promising strategy to destabilize these mineral structures and release the metals. Consequently, microbial leaching technology has emerged as a key research focus for recovering nickel and cobalt from laterite ores, capitalizing on its distinct advantages of being environmentally compatible and cost effective [43].

**Figure 3:**
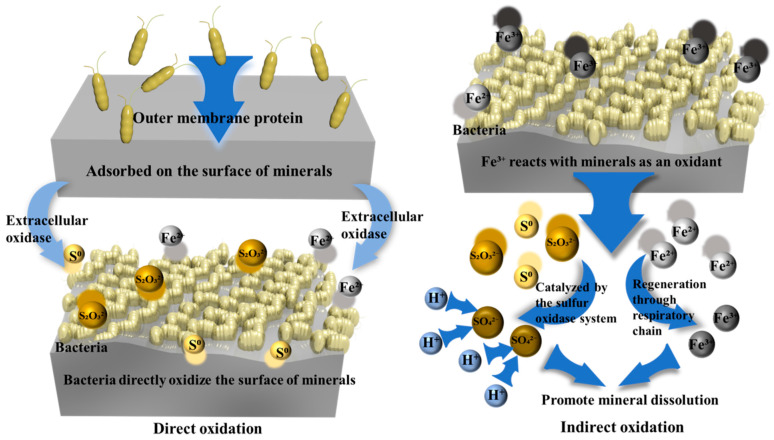
Direct and indirect oxidation mechanisms.

Iron- and sulfur-oxidizing bacteria are a group of acidophiles capable of utilizing both Fe^2+^ and reduced sulfur species (e.g., S^0^, S_2_O_3_^2−^) as dual electron donors for chemolithoautotrophic growth via CO_2_ fixation. They are categorized by temperature tolerance as *mesophilic*, *thermophilic*, or *hyperthermophilic archaea* [44]. These bacteria interact with sulfide minerals through direct and indirect mechanisms (Figure 3). The direct mechanism involves bacterial adhesion to the mineral surface via outer membrane proteins, where extracellular oxidase directly etches the crystal lattice, releasing metal ions and generating S^0^/S_2_O_3_^2−^. The indirect mechanism employs microbially generated Fe^3+^ as a chemical oxidant to attack sulfides. The resulting Fe^2+^ is regenerated to Fe^3+^ through the respiratory chain, while liberated S^0^/S_2_O_3_^2−^ is enzymatically oxidized to SO_4_^2−^ by the sulfur oxidation enzyme system (Sox) [45]. Building upon the fundamental principle of iron-sulfur transformation, this approach can be adapted and specialized for treating specific ores such as laterites. In lateritic ores, valuable metals like cobalt and nickel are often encapsulated within iron (oxy) hydroxide phases (e.g., goethite and arsenic-bearing minerals). Under anaerobic conditions, iron-sulfur oxidizing bacteria (e.g., *Acidithiobacillus ferrooxidans*) can initiate a “reductive dissolution” mechanism. In this process, elemental sulfur serves as the electron donor, reducing structural Fe^3+^ to Fe^2+^ [46]. This reductive reaction effectively disrupts the crystal structure of the hosting minerals. Since cobalt is primarily associated with these phases, this mechanism enables a cobalt leaching recovery exceeding 80% [47]. At the community level, iron- and sulfur-oxidizers form metabolic synergy networks with sulfur-oxidizing bacteria. The Fe^3+^ produced by the former enhances the mineral dissolution kinetics of sulfur-oxidizers, while the continuous acid (H_2_SO_4_) production by sulfur-oxidizers maintains the low pH essential for optimal activity of iron/sulfur oxidation enzymes and Fe^3+^ stability, collectively intensifying leaching efficiency [48]. Furthermore, the introduction of dissimilatory iron-reducing bacteria (DIRB) represents a complementary strategy to enhance the iron cycle described above. DIRB reduce Fe(III) bearing mineral phases (e.g., goethite) into more soluble forms like magnetite or hematite. This reductive transformation disrupts the encapsulating structures that host nickel, thereby creating favorable conditions for subsequent microbial or acid leaching. This approach highlights the remarkable flexibility and application potential of microbial iron sulfur cycling in the environmentally friendly processing of mineral resources [49].

Obligate iron-oxidizing bacteria are chemolithoautotrophic microorganisms utilizing Fe^2+^ as their sole electron donor [50]. They derive energy through Fe^2+^ oxidation catalyzed by the outer-membrane cytochrome c (Cyc2 complex). Representative genera include *Leptospirillum, Ferroplasma,* and *Acidithiobacillus ferridurans*. Compared to iron-sulfur oxidizers, obligatory iron-oxidizers exhibit significant ecological competitiveness for Fe^2+^, stemming from their superior substrate (Fe^2+^) affinity and tolerance to high Fe^3+^ concentrations (maintained via AcrB efflux pumps for iron homeostasis). This makes them pivotal in biomining operations [51]. Their core leaching mechanism relies on an iron cycle. Microbially oxidized Fe^3+^ acts as a chemical oxidant, attacking sulfide minerals, releasing target metal ions and sulfur compounds. The resulting Fe^2+^ is subsequently re-oxidized via the bacterial respiratory chain, establishing a closed-loop regenerative cycle [52].

Obligate sulfur-oxidizing bacteria are chemolithoautotrophs utilizing reduced sulfur compounds (S^0^, S^2−^, S_2_O_3_^2−^, H_2_S) as electron donors, catalyzing energy-yielding metabolism via the sulfur oxidation system (Sox). Key representative genera include *Thermithiobacillus, Acidithiobacillus,* and *Thiomonas.* Sulfur-oxidizers facilitate metal dissolution through synergistic direct and indirect pathways. In the direct mode, cells adsorb onto sulfide surfaces via outer membrane periplasmic copper-containing proteins (e.g., rusticyanin), and membrane-bound thiosulfate oxidases directly catalyze the oxidation of lattice sulfur to SO_4_^2−^, liberating metal ions. In biohydrometallurgical systems, the indirect mechanism mediated by sulfur-oxidizing bacteria serves as the principal pathway for dissolving metals from lateritic nickel ores. The indirect mechanism involves microbial utilization of S^0^/S_2_O_3_^2−^ as substrates for bioacidification, generating high concentrations of H_2_SO_4_ to establish a proton gradient that dissolves minerals [40]. Simultaneously, organic acid anions can form stable, soluble complexes with nickel and cobalt, which significantly enhances their leaching efficiency from the ore matrix [53]. In bioleaching consortia, sulfur-oxidizers frequently operate synergistically with iron-metabolizing bacteria. Within a ternary system (iron-sulfur oxidizers, iron-oxidizers, sulfur-oxidizers), sulfur-oxidizers sustain acidity to promote dissolution. iron-sulfur oxidizers generate the Fe^3+^ oxidant to accelerate sulfide oxidation kinetics. and iron-oxidizers regulate the Fe^3+^/Fe^2+^ ratio, inhibiting the precipitation of passivating secondary minerals like jarosite (KFe_3_(SO_4_)_2_(OH)_6_) on mineral surfaces [54].

As a cornerstone of modern green metallurgy, *acidophilic* microbial leaching technology has reached a high level of maturity in the industrial-scale extraction of metals such as copper, gold, and uranium. Through processes like heap and tank leaching, continuous optimization of bacterial activity and key parameters (e.g., pH, temperature) has significantly enhanced metal recovery from low-grade and refractory ores [55]. However, when applying this technology to lateritic nickel ores, its efficiency becomes highly dependent on the ore’s mineral composition, particularly the occurrence state of nickel and cobalt. Recent mineralogical studies reveal that while nickel hosted within magnesium silicates (e.g., serpentine) can be effectively liberated via microbial acid dissolution, nickel encapsulated within stable goethite lattices results in severely limited bioleaching extraction. This limitation arises because goethite is highly resistant to dissolution under ambient temperature and pressure. Therefore, the judicious selection and combination of acid dissolution and reductive leaching processes are necessary. The recovery of nickel and cobalt from the pregnant leach solution (PLS) is achieved through solvent extraction for purification, using specific extractants like Cyanex 272 (bis(2,4,4-trimethylpentyl) phosphinic acid), followed by metal production via electrowinning [46].

### 3.2. Alkaline-Tolerant Microorganisms and Bioleaching Mechanisms

The depletion of global sulfide ore resources combined with the increasing development of carbonate-type deposits and the pressure to recycle alkaline metallurgical slags collectively drive the expansion of bio-hydrometallurgy into alkaline environments. Conventional *acidophilic* leaching systems face significant limitations under alkaline conditions, including mineral passivation effects and microbial metabolic inhibition. Consequently, research targeting alkaline ore deposits and high-pH tailings has centered on three primary functional groups of alkali-tolerant microbiota including *Pseudomonas genus*, *Actinobacteria phylum* and *Alcaligenes genus* [56].

#### 3.2.1. Pseudomonas Genus

Within the *Pseudomonas genus*, species such as *Pseudomonas putida*, *Pseudomonas fluorescens* and *Pseudomonas azotoformans* demonstrate unique metabolic advantages for metal recovery from low-grade minerals and metallurgical waste, primarily through the secretion of siderophores. *Pseudomonas putida* exhibits the strongest siderophore biosynthetic capability, producing the mixed-type siderophore PyoPpC-3B, containing hydroxamate (-CONHOH) and catechol groups [57]. This molecule facilitates metal liberation via a reduction-dissolution mechanism under neutral to mildly alkaline conditions. The catechol group reduces Fe^3+^ within the mineral lattice of zinc-bearing phases (e.g., willemite, franklinite), disrupting the structure and releasing metal ions like Zn^2+^ and Mn^2+^, while oxidizing itself to o-quinone. This reductive dissolution consumes protons (H^+^), increasing the system pH and thereby accelerating dissolution kinetics [58]. The released Zn^2+^ and Mn^2+^ ions are subsequently chelated by the synergistic action of the hydroxamate and catechol groups, forming stable octahedral complexes. Notably, the complexation efficiency of PyoPpC-3B enhances with rising pH, significantly improving the solution stability of Zn^2+^ and Mn^2+^ [59]. This discovery transcends the traditional understanding of siderophore action relying solely on complexation, revealing for the first time a novel mechanism where the catechol group drives mineral decomposition through a redox cycle.

#### 3.2.2. Actinomycetes

Actinomycetes hold a significant position within the domain of microbial metallurgy, demonstrating considerable potential for the recovery of valuable metals from low-grade ores and tailings. Research indicates that *Nocardiopsis metallicus*, isolated from alkaline metallurgical residues, can directly disrupt the crystal lattice structures of slag materials, such as siliceous alkaline slag, facilitating the release of contained metallic elements [60]. Concurrently, *Nocardiopsis metallicus* secretes metabolites including organic acids (e.g., lactic acid, oxalic acid) and siderophores. These compounds complex with metal ions, forming soluble complexes that promote the transition of metal ions from the solid phase into solution. Furthermore, the bacterial cell surfaces are rich in functional groups (e.g., carboxyl, phosphate groups) capable of adsorbing the dissolved metal ions through ion exchange or electrostatic interactions, achieving in situ enrichment for subsequent recovery. Crucially, *Nocardiopsis metallicus* exhibits strong tolerance to alkaline conditions (pH 7.0–10.5) and high salinity (≤10% NaCl), enabling it to maintain metabolic activity within alkaline industrial waste residues. This capability overcomes the limitations of conventional *acidophilic* leaching microorganisms. The coupled dissolution, adsorption and tolerance mechanism renders this type of actinomycete an efficient biological tool for metal resource recovery from alkaline industrial slags [61].

#### 3.2.3. Alcaligenes Genus

**Figure 4:**
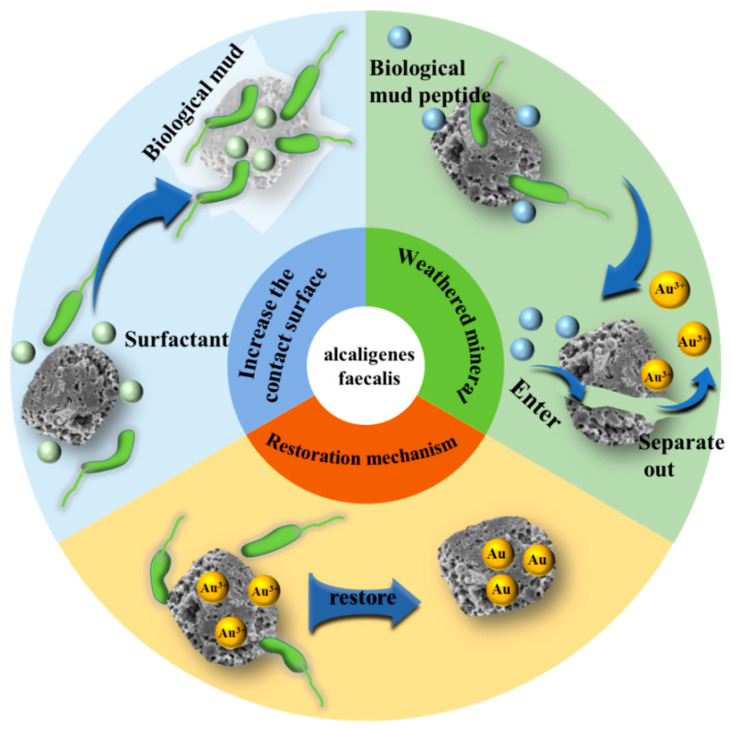
Bioleaching mechanisms of *Alcaligenes faecalis.*

*Alcaligenes faecalis*, a prominent member of the *Alcaligenes genus*, is ubiquitously distributed in soil environments and exhibits robust environmental adaptability [62,63]. It maintains significant viability and physiological activity even under alkaline conditions. Within the field of microbial metallurgy, *Alcaligenes faecalis* demonstrates unique functional characteristics, employing a tripartite synergistic mechanism (Figure 4) to efficiently drive gold ore leaching. Initially, the bacterium secretes surfactants, which reduce the interfacial tension between the leaching agent and the ore, thereby enhancing the detachment of the carbonaceous layer from the mineral surface and optimizing interfacial contact efficiency. Subsequently, the microbially generated extracellular polymeric substances (EPS) penetrate micro-fissures within gold-encapsulating minerals. This penetration induces mineral lattice expansion through bio-oxidative alteration, ultimately liberating encapsulated gold particles. Finally, *Alcaligenes faecalis* possesses the distinctive capability to extracellularly reduce Au^3+^ ions to Au^0^. This inherent reduction process circumvents the need for complex procedures like ultrasonication or the addition of exogenous surfactants to disrupt cell walls for gold recovery, enabling efficient in situ gold reclamation [64].

## 4. Conclusions and Prospects

Alkaline leaching of metallic ores holds significant importance for advancing the sustainable exploitation of mineral resources. Achieving efficient, low-consumption, and eco-friendly metal extraction through the complementary innovation of chemical and biological approaches has become a widely accepted goal in the field. To realize efficient alkaline chemical leaching, selecting appropriate alkaline matrices requires careful consideration of the target ore properties, process economics and environmental constraints, focusing on their reactivity profiles and dissolution characteristics. Incorporating oxidizing agents is an effective strategy to enhance leaching kinetics and selectivity. However, this necessitates balancing their oxidative power, potential reaction by-products and economic viability. Pressure parameters demand precise optimization. Moderate elevation can accelerate extraction by increasing temperature and promoting oxidant solubility, yet excessive levels may reduce effectiveness due to amplified side reactions and passivation layer formation [65]. Bioleaching technology offers dual merits of environmental compatibility and economic efficiency, with its development varying internationally. Currently, research and application of *acidophilic* bioleaching microorganisms are relatively mature [66]. Investigations into alkaliphilic bioleaching microorganisms are still in their nascent stages but demonstrate substantial future promise. *Pseudomonas* species utilize siderophores to solubilize metal ions and also efficiently degrade cyanide compounds [67]. *Actinobacteria* play critical roles in metallurgical slag reclamation and mine site restoration [68]. *Alcaligenes* species exhibit multi-mechanistic interactions during leaching of elemental gold from arsenopyrite. These findings open new technological avenues in biohydrometallurgy, highlighting the extensive research potential within *alkaliphilic* microbial ore processing (Table 1).

Future efforts should intensify systematic research into ore-leaching microorganisms, focusing on deciphering mechanisms governing *alkaliphilic* consortia. Developing synergistic processes combining *alkaliphilic* microorganisms with chemical oxidants is crucial for jointly elevating both the efficiency and quality of metallic ore extraction [69]. Concurrently, technological innovation aimed at lowering production costs and minimizing environmental impacts is essential. This will drive metal ore leaching technology towards greater sustainability and effectiveness, ultimately realizing the responsible development and utilization of mineral resources.

## Figures and Tables

**Table 1:** Summary of chemical alkaline leaching and biological leaching.

Serial Number	Types of Substances and Bacterial Strains	Property Classification	Oxidizable Metal	References
1	Sodium hydroxide or potassium hydroxide	Alkaline matrix	Zinc oxide ore, stibnite	[9,10]
2	Glycine	Alkaline matrix	Cuprite, Low-sulfide oxidized gold ores	[11,12]
3	Sodium sulfide	Alkaline matrix	Lead-zinc ores, Tetrahedrite-rich concentrates containing copper and antimony	[16,17]
4	Sodium hypochlorite	Alkaline matrix	Copper concentrates, tennantite, Gold-bearing enargite	[19,20,21]
5	Hydrogen peroxide	Oxidants	Chromium-containing slag	[23,24,25]
6	Ozone	Oxidants	Pyrite, Uranium ore	[27,28]
7	Oxygen	Oxidants	Manganese ore, Arsenic-bearing residues	[31,32]
8	Potassium permanganate	Oxidants	copper sulfide ores	[12,33]
9	Iron- and sulfur-oxidizing bacteria	*Acidophilic* microorganisms	Sulfide minerals, laterite ore	[44,45,46,47,49]
10	Obligate iron-oxidizing bacteria	*Acidophilic* microorganisms	Sulfide minerals	[50,51,52]
11	Obligate sulfur-oxidizing bacteria	*Acidophilic* microorganisms	Sulfide minerals, laterite ore	[40,53,54]
12	*Pseudomonas genus*	*Alkaline-tolerant* microorganisms	Zinc-containing minerals	[57,58,59]
13	*Actinomycetes*	*Alkaline-tolerant* microorganisms	siliceous alkaline slag	[60,61]
14	*Alcaligenes genus*	*Alkaline-tolerant* microorganisms	gold ore	[62,63,64]

## Data Availability

No new data were created or analyzed in this study. Data sharing is not applicable to this article.

## References

[1] Hasan M.A., Hossain R., Sahajwalla V. (2023). Critical metals (Lithium and Zinc) recovery from battery waste, ores, brine, and steel dust: A review. Process Saf. Environ. Prot..

[2] Saidi A., El Khawaja R., Boffito D.C. (2023). A Review of Traditional and Intensified Hydrometallurgy Techniques to Remove Chromium and Vanadium from Solid Industrial Waste. ACS Eng. Au.

[3] Hubau A., Guezennec A.G., Joulian C., Falagán C., Dew D., Hudson-Edwards K.A. (2020). Bioleaching to reprocess sulfidic polymetallic primary mining residues: Determination of metal leaching mechanisms. Hydrometallurgy.

[4] Ma A.Y., Zheng X.M., Gao L., Li K.Q., Omran M., Chen G. (2022). Investigations on the Thermodynamics Characteristics, Thermal and Dielectric Properties of Calcium-Activated Zinc-Containing Metallurgical Residues. Materials.

[5] Chiang Y.W., Santos R.M., Monballiu A., Ghyselbrecht K., Martens J.A., Mattos M.L.T., Van Gerven T., Meesschaert B. (2013). Effects of bioleaching on the chemical, mineralogical and morphological properties of natural and waste-derived alkaline materials. Miner. Eng..

[6] Zhang Q., Zheng X.H., Lv W.G., He M.M., Yan W.Y., Gao W.F., Ning P.G., Cao H.B., Sun Z. (2025). An acid-free process to prepare battery grade nickel and cobalt sulfates from complex resources. Nat. Commun..

[7] Wang X.X., Li J.H., Sun P., Deng Z.G., Li X.B., Li M.T., Wei C. (2025). Removing fluorine and chlorine from zinc oxide dust by wet alkaline washing and studying fluorine occurrence states. Int. J. Chem. React. Eng..

[8] Jia L.P., Huang J.J., Ma Z.L., Liu X.H., Chen X.Y., Li J.T., He L.H., Zhao Z.W. (2020). Research and development trends of hydrometallurgy: An overview based on *Hydrometallurgy* literature from 1975 to 2019. Trans. Nonferrous Met. Soc. China.

[9] Zhao Y.C., Stanforth R. (2000). Production of Zn powder by alkaline treatment of smithsonite Zn-Pb ores. Hydrometallurgy.

[10] Zekavat M., Yoozbashizadeh H., Khodaei A. (2021). Leaching of Antimony from Stibnite Ore in KOH Solution for Sodium Pyroantimonate Production: Systematic Optimization and Kinetic Study. JOM.

[11] Tanda B.C., Eksteen J.J., Oraby E.A. (2017). An investigation into the leaching behaviour of copper oxide minerals in aqueous alkaline glycine solutions. Hydrometallurgy.

[12] Oraby E.A., Eksteen J.J., O’Connor G.M. (2020). Gold leaching from oxide ores in alkaline glycine solutions in the presence of permanganate. Hydrometallurgy.

[13] Barragán-Mantilla S.P., Gascó G., Almendros P., Méndez A. (2024). Insights into the use of green leaching systems based on glycine for the selective recovery of copper. Miner. Eng..

[14] Huang Y.K., Wang D.S., Liu H.T., Fan G.X., Peng W.J., Cao Y.J. (2023). Selective complexation leaching of copper from copper smelting slag with the alkaline glycine solution: An effective recovery method of copper from secondary resource. Sep. Purif. Technol..

[15] Oraby E., Li H., Deng Z.X., Eksteen J. (2023). Selective extraction of Ni and Co from a pyrrhotite-rich flotation slime using an alkaline glycine-based leach system. Miner. Eng..

[16] Aghazadeh S., Abdollahi H., Gharabaghi M., Mirmohammadi M. (2021). Selective leaching of antimony from tetrahedrite rich concentrate using alkaline sulfide solution with experimental design: Optimization and kinetic studies. J. Taiwan Inst. Chem. Eng..

[17] Guo X.Y., Xu Z.P., Li D., Tian Q.H., Xu R.Z., Zhang Z. (2017). Recovery of tellurium from high tellurium-bearing materials by alkaline sulfide leaching followed by sodium sulfite precipitation. Hydrometallurgy.

[18] Liu R.Q., Zhong C.Y., Lin S.Y., He D.D., Sun W. (2022). Depression Mechanism of Sodium Sulfide in Flotation Separation of Molybdenite and Bismuthinite. Miner. Process. Extr. Metall. Rev..

[19] Liu Y., Zhong H., Cao Z. (2010). Molybdenum removal from copper ore concentrate by sodium hypochlorite leaching. Min. Sci. Technol..

[20] Hernández M.C., Benavente O., Roca A., Melo E., Quezada V. (2023). Selective Leaching of Arsenic from Copper Concentrates in Hypochlorite Medium. Minerals.

[21] Curreli L., Ghiani M., Surracco M., Orrù G. (2005). Beneficiation of a gold bearing enargite ore by flotation and As leaching with Na-hypochlorite. Miner. Eng..

[22] Montoya A., Reyes J.L., Reyes I.A., Cruz R., Lázaro I., Rodríguez I. (2023). Effect of sodium hypochlorite as a depressant for copper species in Cu-Mo flotation separation. Miner. Eng..

[23] Peng H., Guo J., Lv L., Huang H., Li B. (2020). Recovery of chromium by calcium-roasting, sodium-roasting, acidic leaching, alkaline leaching and sub-molten technology: A review. Environ. Chem. Lett..

[24] Nicol M.J. (2020). The role and use of hydrogen peroxide as an oxidant in the leaching of minerals. II. alkaline solutions. Hydrometallurgy.

[25] Nwaila G.T., Notole V., Alex S., Ghorbani Y. (2025). Optimizing Gold Recovery from Witwatersrand-Type Ores Using Alkaline Glycine Leaching and Conditional Simulation. Nat. Resour. Res..

[26] Suyantara W.G.P., Berdakh D., Miki H., Hirajima T., Sasaki K., Ochi D., Aoki Y. (2023). Effect of hydrogen peroxide on selective flotation of chalcocite and enargite. Int. J. Min. Sci. Technol..

[27] Trujic S., Popovic M.P., Conic V., Janosevic M., Alimpic F., Bajic D., Milenkovic-Andelkovic A., Abramovic F. (2025). Ozone/Thiosulfate-Assisted Leaching of Cu and Au from Old Flotation Tailings. Molecules.

[28] Zhang R., Hou W., Wang H.Q., Hu E.M., Lei Z.W., Hu F., Zhou W., Wang Q.L. (2022). Oxidative leaching of sandstone uranium ore assisted by ozone micro-nano bubbles. J. Radioanal. Nucl. Chem..

[29] Hunter E. (2013). On the Leaching Behavior of Uranium-Bearing Resources in Carbonate-Bicarbonate Solution by Gaseous Oxidants. Ph.D. Thesis.

[30] Wang J.X., Faraji F., Ghahreman A. (2021). Evaluation of ozone as an efficient and sustainable reagent for chalcopyrite leaching: Process optimization and oxidative mechanism. J. Ind. Eng. Chem..

[31] Luo F.L., Xie H.Y., Jin H.X., Li C.Z., Ma L.R., Wang D.L. (2023). Oxidative Leaching of Low-Grade Pyrolusite in Alkaline Solutions to Produce Potassium Manganate. Min. Metall. Explor..

[32] Liu W., Huang C., Han J.W., Qin W.Q. (2021). Removal and reuse of arsenic from arsenic-bearing purified residue by alkaline pressure oxidative leaching and reduction of As (V). Hydrometallurgy.

[33] Guo S.H., Kuang J.Z., Wang L. (2025). Interfacial reaction mechanisms of potassium permanganate and sodium persulfate in separating stibnite from arsenopyrite. Surf. Interfaces.

[34] Wang M.S., Wei C., Fan G., Li M.T., Deng Z.G., Wang S.F. (2015). Selective extraction of Mo from a Ni-Mo ore using pressure alkaline leaching. Hydrometallurgy.

[35] Jiang L.S., Leng H.G., Han B.S. (2023). Dissolution and Passivation Mechanism of Chalcopyrite during Pressurized Water Leaching. Minerals.

[36] McDonald R.G., Muir D.M. (2007). Pressure oxidation leaching of chalcopyrite Part II: Comparison of medium temperature kinetics and products and effect of chloride ion. Hydrometallurgy.

[37] Petersen J. (2023). From understanding the rate limitations of bioleaching mechanisms to improved bioleach process design. Hydrometallurgy.

[38] Nnaemeka I.C., O C.T., Nonso U.C., Ikechukwu O.M., Anezichukwu A.F., Ikechukwu N.A., M O., Chukwudi E.B., Chisom M.K., Ifeanyichukwu O.T. (2025). Examining the efficiency of microbe-assisted metal extraction: A review of bio-hydrometallurgical leaching techniques. Hybrid J..

[39] Jia B.J., Yu J.M. (2012). The Research Status and Development Trend of Microbial Flocculant. Phys. Procedia.

[40] Nguyen V.K., Lee M.H., Park H.J., Lee J.U. (2015). Bioleaching of arsenic and heavy metals from mine tailings by pure and mixed cultures of *Acidithiobacillus*, spp.. J. Ind. Eng. Chem..

[41] Roberto F.F., Schippers A. (2022). Progress in bioleaching: Part B, applications of microbial processes by the minerals industries. Appl. Microbiol. Biotechnol..

[42] Jones S., Santini J.M. (2023). Mechanisms of bioleaching: Iron and sulfur oxidation by acidophilic microorganisms. Essays Biochem..

[43] Behera S.K., Mulaba-Bafubiandi A.F. (2015). Advances in microbial leaching processes for nickel extraction from lateritic minerals—A review. Korean J. Chem. Eng..

[44] Crundwell F.K. (2003). How do bacteria interact with minerals?. Hydrometallurgy.

[45] Koizhanova A., Magomedov D., Abdyldayev N., Kamalov E., Yerdenova M., Bakrayeva A. (2022). Copper Extraction from Complex Waste Dumps by Biochemical Leaching Method. J. Ecol. Eng..

[46] Marrero J., Coto O., Goldmann S., Graupner T., Schippers A. (2015). Recovery of Nickel and Cobalt from Laterite Tailings by Reductive Dissolution under Aerobic Conditions Using *Acidithiobacillus* Species. Environ. Sci. Technol..

[47] Stanković S., Martin M., Goldmann S., Gäbler H.-E., Ufer K., Haubrich F., Moutinho V.F., Giese E.C., Neumann R., Stropper J.L. (2022). Effect of mineralogy on Co and Ni extraction from Brazilian limonitic laterites via bioleaching and chemical leaching. Miner. Eng..

[48] Chen Z., Huang X., He H., Tang J., Tao X., Huang H., Haider R., Ali M.I., Jamal A., Huang Z. (2021). Bioleaching Coal Gangue with a Mixed Culture of *Acidithiobacillus ferrooxidans* and *Acidithiobacillus thiooxidans*. Minerals.

[49] Sukla L.B., Pattanaik A., Samal D.P.K., Pradhan D. (2021). Microbial Leaching for Recovery of Nickel and Cobalt from Lateritic Ore: A Review. Ni-Co 2021: The 5th International Symposium on Nickel and Cobalt. The Minerals, Metals & Materials Series.

[50] Kato S., Ohkuma M. (2021). A Single Bacterium Capable of Oxidation and Reduction of Iron at Circumneutral pH. Microbiol. Spectrum.

[51] Liu R.H., Zhou H.B. (2022). Growth in ever-increasing acidity condition enhanced the adaptation and bioleaching ability of *Leptospirillum ferriphilum*. Int. Microbiol..

[52] Pakostova E., Grail B.M., Johnson D.B. (2017). Indirect oxidative bioleaching of a polymetallic black schist sulfide ore. Miner. Eng..

[53] Hetz S.A., Schippers A. (2025). Aerobic Bioleaching of Six Brazilian Laterite Ores with *Acidithiobacillus thiooxidans*, *Sulfobacillus* species and *Archaea* at Various Conditions. J. Sustain. Metall..

[54] Gu G.-H., Hu K.-T., Li S.-K. (2013). Bioleaching of chalcopyrite by *Leptospirillum ferriphilum*. J. Cent. South Univ..

[55] López-Martínez A., Martínez-Prado M.A., Núñez-Ramírez D.M., Medina-Torres L., Rojas-Contreras J.A., Anguiano-Vega G.A., Soto-Cruz N.O. (2025). Acidophilic bacteria for metal extraction: Biotechnological characteristics and applications. Braz. J. Chem. Eng..

[56] Dey S. (2022). Microbial Resources of Alkaline Bauxite Residue and Their Possible Exploitation in Remediation and Rehabilitation. Geomicrobiol. J..

[57] Han P., Liu T., Zheng Y., Song R., Nan T., Yang X., Huang L., Yuan Y. (2022). A Mycorrhizal Bacteria Strain Isolated From Polyporus umbellatus Exhibits Broad-Spectrum Antifungal Activity. Front. Plant Sci..

[58] Williamson A.J., Folens K., Matthijs S., Cortes Y.P., Varia J., Du Laing G., Boon N., Hennebel T. (2021). Selective metal extraction by biologically produced siderophores during bioleaching from low-grade primary and secondary mineral resources. Miner. Eng..

[59] Boguta P., Sokołowska Z. (2020). Zinc Binding to Fulvic acids: Assessing the Impact of pH, Metal Concentrations and Chemical Properties of Fulvic Acids on the Mechanism and Stability of Formed Soluble Complexes. Molecules.

[60] Shivlata L., Satyanarayana T. (2015). Thermophilic and alkaliphilic *Actinobacteria*: Biology and potential applications. Front. Microbiol..

[61] Schippers A., Bosecker K., Willscher S., Spröer C., Schumann P., Kroppenstedt R.M. (2002). *Nocardiopsis metallicus* sp. nov., a metalleaching actinomycete isolated from an alkaline slag dump. Int. J. Syst. Evol. Microbiol..

[62] Lwin H.W., Dattelbaum J.D. (2025). Isolation and Whole Genome Sequence Analysis of *Alcaligenes* and *Chromobacterium* Strains with Antimicrobial Activity Against ESKAPE Pathogen Relatives. J. Genom..

[63] Eltokhy M.A., Saad B.T., Eltayeb W.N., El-Ansary M.R., Aboshanab K.M., Ashour M.S.E. (2021). A Metagenomic Nanopore Sequence Analysis Combined with Conventional Screening and Spectroscopic Methods for Deciphering the Antimicrobial Metabolites Produced by *Alcaligenes faecalis* Soil Isolate MZ921504. Antibiotics.

[64] Pineda Y.S., Devries S.L., Steiner N.C., Block-Cora K.A. (2023). Bioleaching of Gold in Mine Tailings by *Alcaligenes faecalis*. Minerals.

[65] Whitworth A.J., Vaughan J., Southam G., Van der Ent A., Nkrumah P.N., Ma X.D., Parbhakar-Fox A. (2022). Review on metal extraction technologies suitable for critical metal recovery from mining and processing wastes. Miner. Eng..

[66] Mishra S., Panda S., Akcil A., Dembele S., Agcasulu I. (2021). A Review on Chemical versus Microbial Leaching of Electronic Wastes with Emphasis on Base Metals Dissolution. Minerals.

[67] Moradkhani M., Yaghmaei S., Nejad Z.G. (2018). Biodegradation of Cyanide under Alkaline Conditions by a Strain of Pseudomonas putida Isolated from Gold Mine Soil and Optimization of Process Variables through Response Surface Methodology (RSM). Period. Polytech.-Chem. Eng..

[68] Newsome L., Falagán C. (2021). The Microbiology of Metal Mine Waste: Bioremediation Applications and Implications for Planetary Health. GeoHealth.

[69] Kara I.T., Kremser K., Wagland S.T., Coulon F. (2023). Bioleaching metal-bearing wastes and by-products for resource recovery: A review. Environ. Chem. Lett..

